# The patient journey to diagnosis and treatment of autoinflammatory diseases

**DOI:** 10.1186/s13023-018-0902-7

**Published:** 2018-09-06

**Authors:** Jonathan S. Hausmann, Kathleen G. Lomax, Ari Shapiro, Karen Durrant

**Affiliations:** 1 Boston Children’s Hospital, Boston, MA 02115 USA; 2 Beth Israel Deaconess Medical Center, Boston, MA 02215 USA; 3 Novartis Pharmaceuticals Corporation, East Hanover, NJ USA; 4 Flince Research, Brooklyn, NY USA; 5 Autoinflammatory Alliance, San Francisco, CA USA

**Keywords:** Autoinflammatory diseases, Patient journey, Disease experience, Psycho-social dynamics, Parent experience

## Abstract

**Background:**

Limited data are available on the experiences of patients with autoinflammatory diseases (AIDs) and their families along the path to diagnosis and treatment. We sought to describe these experiences in patients with AIDs including tumor necrosis factor receptor-associated periodic syndrome (TRAPS), mevalonate kinase deficiency/hyperimmunoglobulin D syndrome (MKD/HIDS), and familial Mediterranean fever (FMF).

**Methods:**

Ninety-minute, semi-structured qualitative interviews and 5-day written/video diaries were used to gather information on the experiences of patients with AIDs and their families.

**Results:**

Twelve families of patients from the US (TRAPS [*n* = 4], MKD/HIDS [*n* = 5], FMF [*n* = 5]) participated in this study from August to November 2015. The study included two families with multiple afflicted siblings. Patients’ ages ranged from 1 to 28 years. Most parents reported realizing that something was seriously wrong with their child after medical emergencies and/or hospitalizations, which initiated the difficult path to diagnosis. For most, the process included multiple specialist visits, extensive and repeated testing, and many misdiagnoses. Over time, 92% of parents reported losing confidence in the healthcare system’s ability to find an answer to their child’s symptoms, while they also struggled with unsupportive school personnel and dismissive friends and relatives. Patients and their parents reported holding on to memories of “what life was like” before the onset of symptoms and mourning their subsequent loss of “normalcy.” Even after diagnosis, patients and parents continued to feel uncertain about what to expect in the future.

**Conclusions:**

All families emphasized the need for efficient early diagnosis of AIDs. Initiatives that improve the speed and accuracy of diagnosis, provide more comprehensive patient education, and support patients and families through the illness have the potential to significantly improve the quality of life of patients with AIDs and their families. Healthcare providers should be aware of the impact of the long diagnostic journey on families and work to create an environment of trust and collaboration in the face of a difficult and prolonged diagnostic process.

**Electronic supplementary material:**

The online version of this article (10.1186/s13023-018-0902-7) contains supplementary material, which is available to authorized users.

## Background

Periodic fever syndromes, such as familial Mediterranean fever (FMF), mevalonate kinase deficiency/hyperimmunoglobulin D syndrome (MKD/HIDS), and tumor necrosis factor (TNF) receptor-associated periodic syndrome (TRAPS), are rare autoinflammatory conditions characterized by recurrent fevers and systemic inflammation [1]. These disorders are challenging to diagnose because their symptoms are nonspecific and resemble other infectious and malignant diseases. Although provisional classification criteria have been developed to help clinicians to diagnose these rare syndromes [2], patients often experience delays in diagnosis ranging from months to years, which can lead to additional morbidity [3–6]. To date, the experiences of such patients and their families along the path to diagnosis have been poorly studied. The objective of this study was to define the stages of the patient journey by following the experiences of patients with AIDs and their families over the time through diagnosis and treatment.

## Methods

Patients with AIDs and their families were recruited by physicians with expertise in AIDs and through the Autoinflammatory Alliance, an AIDs patient support group. Researchers used qualitative, in-depth, semi-structured telephone interviews (Interview guide is available online as Additional file 1) and 5-day written and video diaries to explore the history and daily lives of these families. This was a market research study and therefore no ethics committee approval was required. All patients provided written informed consent to participate.

## Results

### Patient and family characteristics

Twelve families of patients with autoinflammatory diseases participated in this study from August to November 2015. Families included 4 patients with TRAPS, 5 with MKD/HIDS, and 5 with FMF (*n* = 5); two families had multiple afflicted siblings. Patients’ ages ranged from 1 to 28 years and all were from the United States. The time to diagnosis varied from 2.5–24 years; in patients whose siblings or other family members had already been diagnosed, the time to diagnosis was shorter. The age (median [min- max]) at diagnosis was 4 (3–10), 4.5 (1–15) and 3 (2–16) years for TRAPS, FMF and HIDS patients, respectively. Patients were currently receiving or had previously been treated with one or more of the following medications: colchicine, anakinra, canakinumab, tocilizumab, and anti-TNF agents (etanercept, adalimumab, and certolizumab).

### Parents’ perceptions pre-diagnosis

**Fig. 1:**
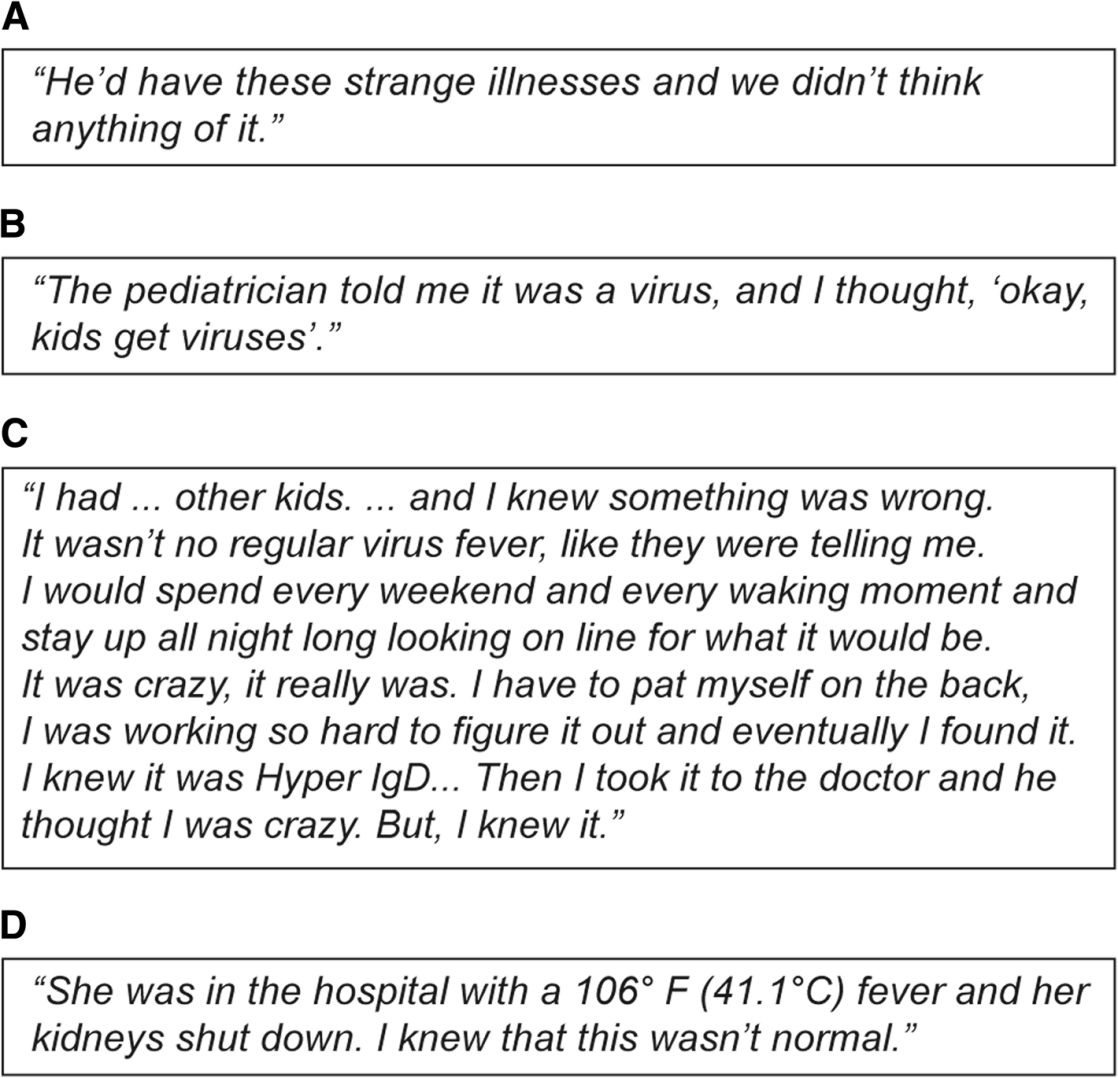
Quotes from parents of children with AIDs on difficulties experienced prior to receiving a diagnosis. Parent perceptions included: **a** Their child had an intermittent illness; **b** They were given simple explanations from doctors that were misdiagnoses; **c** They suffered from increased anxiety with worsening of their child’s illness; **d** They struggled to find answers. AIDs, autoinflammatory diseases

Children with AIDs in this study appeared healthy during their early childhood, with most meeting early developmental milestones. Childhood illnesses emerged but rarely raised the alarm for the parents (Fig. 1a)*.* Owing to the nonspecific nature of the symptoms, physicians tended to look for easily explainable causes such as recurrent viral illnesses. Many parents reported receiving simple explanations from their physicians that later turned out to be misdiagnoses (Fig. 1b). Worsening patterns of illness over time increased parents’ anxiety, and they became increasingly fearful and confused. Figure 1c provides an example of one such parent who stopped accepting her pediatrician’s explanations and began looking for her own answers to her child’s symptoms. First-time parents indicated that they had an especially difficult time recognizing that their child’s symptoms could be more than a common viral illness. Most parents (86%) reported that they only realized that something was seriously wrong with the health of their child after a severe medical emergency and/or hospitalization (Fig. 1d). Furthermore, many patients and their parents reported that they held on to their memories of what life was like before the onset of symptoms and mourned their subsequent loss of “normalcy.”

### Difficulties in establishing the diagnosis of AIDs

**Fig. 2:**
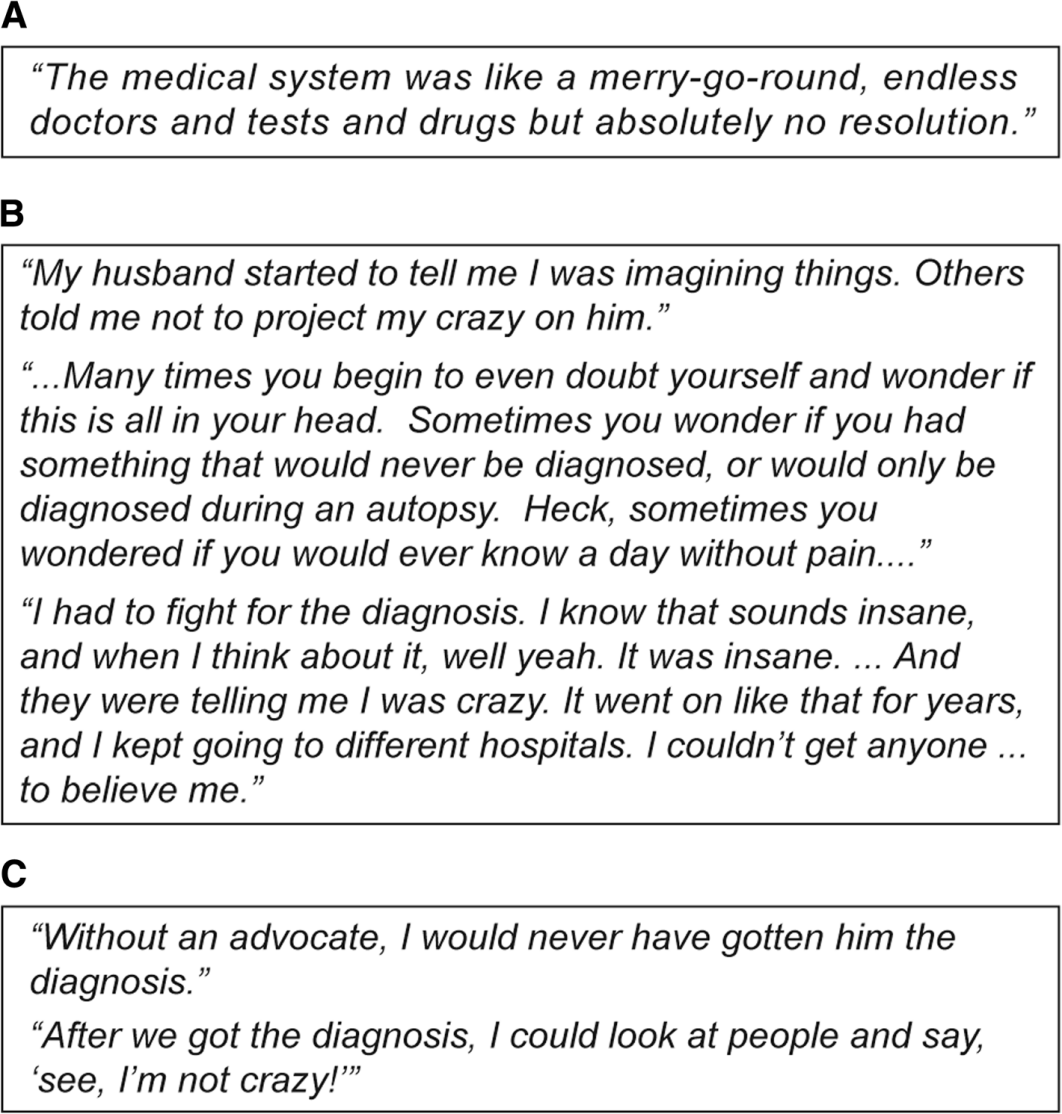
Quotes from parents of children with AIDs on the difficulties experienced during diagnosis. Challenges experienced during diagnosis included: **a** Parents encounter the medical “merry-go-round”; **b** Parents encounter unsympathetic voices on the path to diagnosis; **c** Families required a physician advocate to get their child diagnosed. AIDs, autoinflammatory diseases

Parents encountered medical “merry-go-rounds” involving many specialist visits and diagnostic tests (Fig. 2a). For many, the diagnostic path included several specialist visits, long wait times for appointments, extensive testing, and misdiagnoses including Lyme disease, meningitis, H1N1 influenza, systemic lupus erythematosus, systemic juvenile idiopathic arthritis, atypical Kawasaki’s disease, leukemia, lymphoma, bone cancer, and Crohn’s disease. The most common misdiagnoses by general pediatricians included common cold, food allergy, hay fever, and varicella. Payors and health plans for several patients limited specialist visits, explaining that fevers are not notable causes of long-term damage, as well as the absence of confirmed diagnoses. Most parents (92%) reported losing confidence in the healthcare system’s ability to find an answer to their child’s symptoms, while they also struggled with unsupportive school personnel and dismissive friends and relatives. Many parents stated that they lost self-confidence and began doubting their own judgment as it pertained to their child’s health (Fig. 2b). The feeling of lost confidence was further compounded by frustrations caused by an arduous and inconclusive diagnostic process. Many parents (generally mothers) began to question their own mental health and wondered if the symptoms were indeed real or if they were imagining things. As shown in Fig. 2c, some parents had to become their own advocate, and most parents felt that a physician advocate (a particularly engaged and proactive pediatrician, pediatric nurse, or pediatric rheumatologist) helped them to arrive at a diagnosis.

### The emotional impact of AIDs on children and their families

**Fig. 3:**
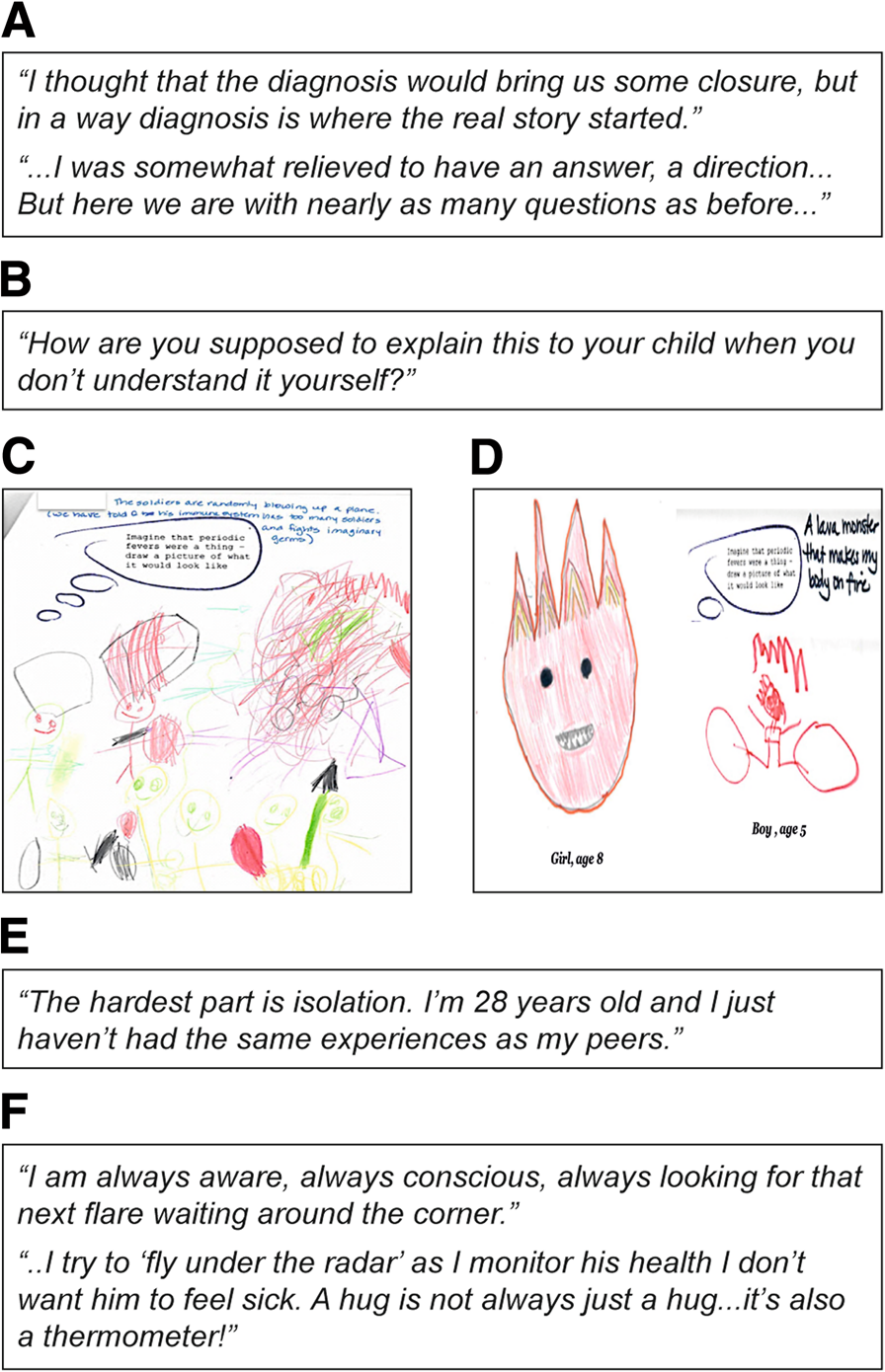
Quotes from parents of children and drawing from patients with AIDs on emotional aspects. Emotional impact on the patients and their parents included: **a** After diagnosis, several questions remained unanswered from the parents’ perspective; **b** Parents had difficulty in explaining the disease to their children; **c** Patient’s perception of their mother’s explanation of HIDS: “Soldiers in your body fight imaginary germs;” **d** Drawings by patients with AIDs in response to how they perceive their disease; **e** Children with AIDs and their families experienced overwhelming isolation; **f** Parents remained hypervigilant about their child’s health. HIDS, hyperimmunoglobulin D syndrome; AIDs, autoinflammatory diseases

While an accurate diagnosis provided vindication and relief, parents reported that it also led to further unanswered questions (Fig. 3a). Figure 3b shows that some parents had difficulties explaining the disease to their children in an age-appropriate manner. Some children with AIDs drew monsters when asked to depict how they felt about their disease (Fig. 3c, d).

Children with AIDs and their families experienced isolation (Fig. 3e). Parents reported that they remained hypervigilant (Fig. 3f) regarding their child’s health and lived in constant anticipation of the next flare, irrespective of the success of their current therapy. Parents perceived the next flare as being “always around the corner.”

### Parents’ and patients’ experiences of daily symptoms

**Fig. 4:**
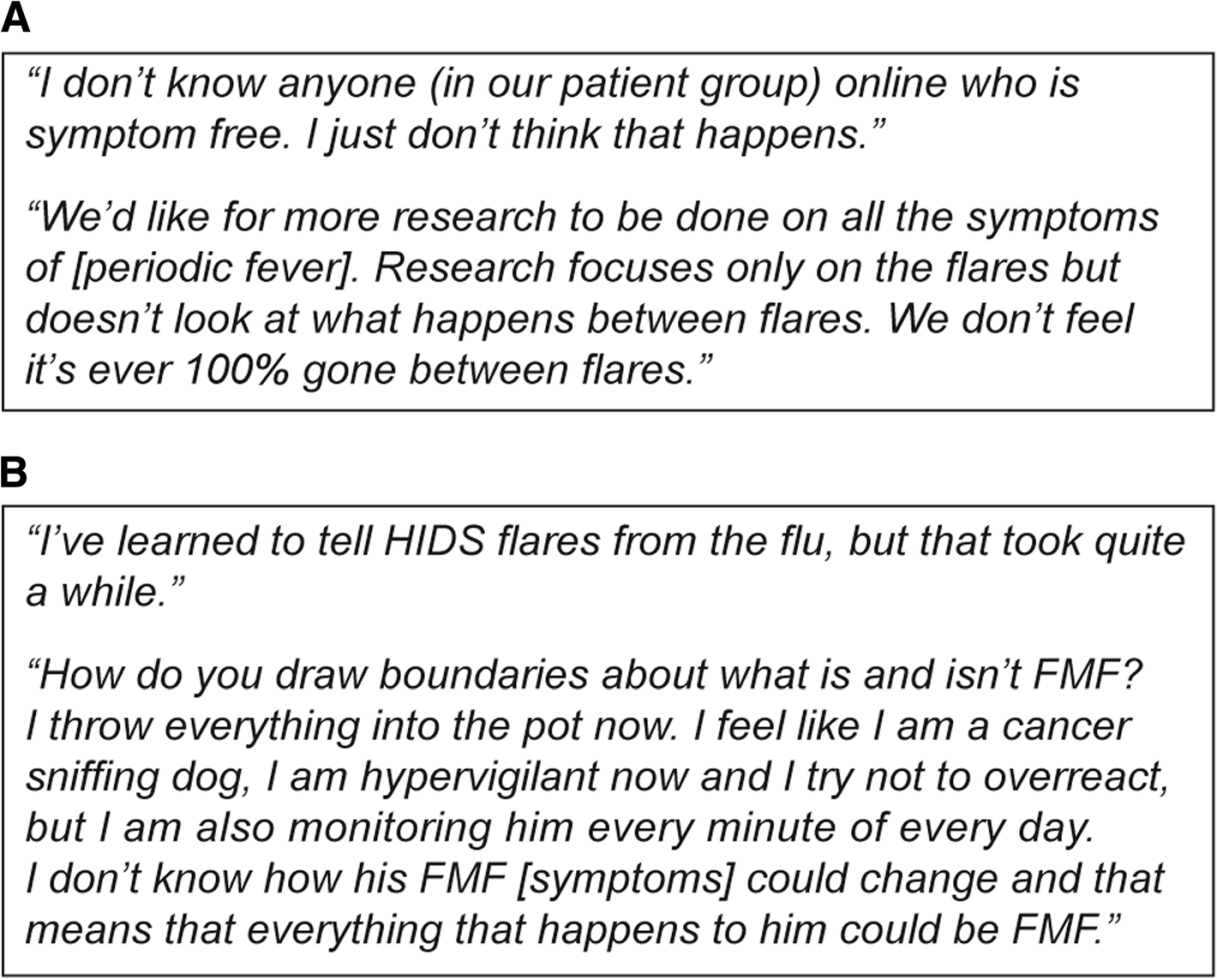
Quotes from parents of children and patients with AIDs on the difficulties experienced post-diagnosis. Difficulties encountered by parents and patients included: **a** Some patients experienced daily symptoms; **b** Parents struggled to differentiate routine childhood illnesses from AIDs. FMF, familial Mediterranean fever; HIDS, hyperimmunoglobulin D syndrome; AIDs, autoinflammatory diseases

Many patients (64%) reported the presence of disease symptoms between flares (Fig. 4a) despite receiving medications. These included myalgias, lymphadenopathy, aphthous ulcers, fatigue, and gastrointestinal symptoms. Daily symptoms persisted in some patients irrespective of flares and independent of fevers. Parents expressed little hope of improving these daily symptoms and reported that they were frustrated with physicians’ inability to acknowledge symptoms between disease flares, which the parents considered to be related to the disease. Also, children with AIDs continued to suffer from common childhood illnesses, and many parents struggled to differentiate these from AID flares (Fig. 4b). Parents also commented that physicians in the emergency department, primary care providers, and other specialists also struggled to assess the condition of their children. Many parents developed an aversion to seeking medical care from providers who lacked education around AIDs and their management.

### Uncertainty post-diagnosis

**Fig. 5:**
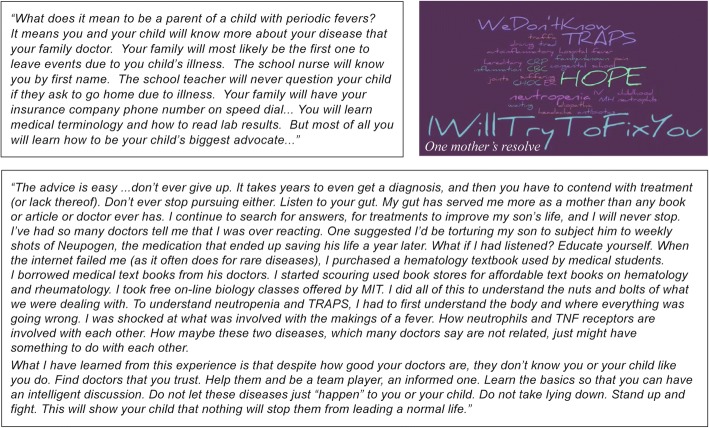
Quotes from mothers of children with AIDs on the responsibilities taken for their child’s care. AIDs, autoinflammatory diseases

**Fig. 6:**
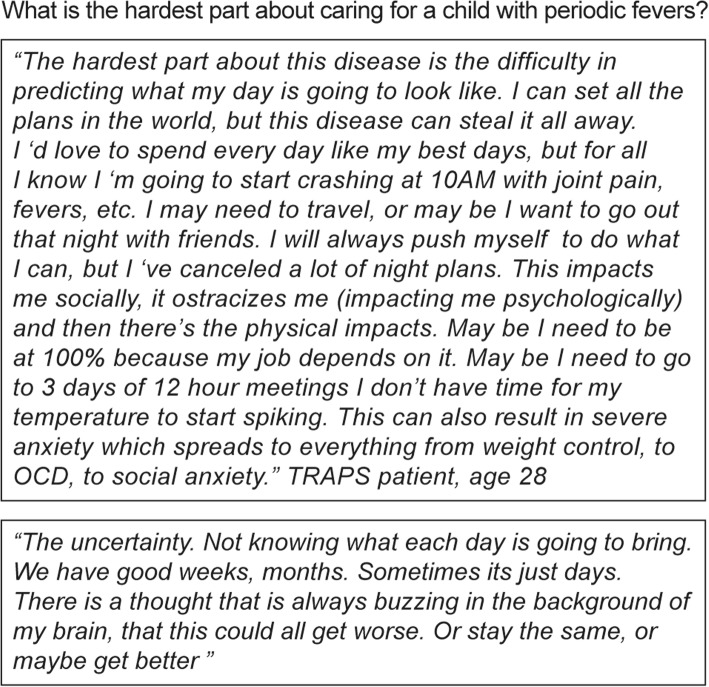
Quotes from children with AIDs and their parents on the uncertainty that pervades their lives. AIDs, autoinflammatory diseases

Parents reported the need to coordinate the process of care between their child’s pediatrician, specialists, school nurses, and others (Fig. 5). After diagnosis, many parents concluded that the feelings of uncertainty was a hallmark of AIDs, which was one of the most emotionally challenging factors for them and their children (Fig. 6).

## Discussion

In this study, we analyzed interview reports and written and video diaries from 12 families of patients with AIDs to understand their experiences from the onset of symptoms to diagnosis and treatment. The present qualitative analysis demonstrated that the burden of AIDs is considerable, and impacts the physical, social, and emotional aspects of patients and their families. AIDs are often misdiagnosed, leading to unnecessary hospitalizations and therapeutic procedures [7–9]. In our study, we observed that patients with AIDs encountered a notable delay in the time to diagnosis that led to considerable stress and confusion for patients and their families.

One of the most difficult aspects expressed by parents of children with AIDs was the loss of what they considered a “normal” life, and this realization was the core tragedy for several families. Parents also reported feeling a loss of self-confidence with increasing alienation in the face of criticism and disbelief. Such experiences led to distrust in medical establishments, which persisted even after diagnosis.

A definitive diagnosis of AIDs provided vindication and relief, as well as an impetus to focus on further education and treatment options, yet it still took several parents a long time to trust the healthcare system again. Reports of distress and confusion during the diagnostic process have been documented in many families with children suffering from other disorders, including congenital heart defects and cleft palate [10, 11]. In this study, uncertainty was reported as a cornerstone of life with AIDs. Uncertainities about the diagnosis and its implications led familiies to seek for online support and community.

Healthcare providers should be aware of the impact of the prolonged diagnostic journey on families and work to create an environment of trust where families and clinical staff can work collaboratively.

These findings should be interpreted in light of the qualitative nature of the study and the small sample size. All patients were from the United States, and so our findings may not reflect the diagnostic experience of patients with AIDs from other countries.

## Conclusions

Our work highlights the need for an efficient and timely diagnosis of AIDs. Because the field of autoinflammation is relatively new and the diseases are rare, it is important to educate primary care providers to recognize these diseases among the many that cause frequent fevers. Providers should learn how to conduct the initial workup and when to refer patients with suspected AIDs to rheumatologists experienced in diagnosing and managing these rare syndromes [1]. Autoinflammatory Disease Clinics are being established as referral centers for this purpose. For patients and their families, patient support networks provide emotional support, advice in managing the medical and psychosocial aspects of AIDs, as well as tools to help prepare families for the future  (a list of useful resources for patients and medical professionals are found in the Additional file 1). Treatment strategies also need to offer consistent and comprehensive patient education and support for patients and their families. Research to expedite diagnosis (such as with the use of wearable thermometers) and to enhance the recognition and treatment of daily symptoms that patients experience, could significantly improve the lives of patients with AIDs and their families.

## Additional file

Additional file 1: Interview Guide and Resources for Patients and Medical Professionals. (DOCX 44 kb)

## Abbreviations

AIDs: Autoinflammatory diseases
FMF: Familial Mediterranean fever
MKD/HIDS: Mevalonate kinase deficiency/hyperimmunoglobulin D syndrome
TNF: Tumor necrosis factor
TRAPS: Tumor necrosis factor receptor-associated periodic syndrome
