# Supplementary material for: The patient journey to diagnosis and treatment of autoinflammatory diseases
Source: Orphanet J Rare Dis. 2018 Sep 6;13:156. doi: 10.1186/s13023-018-0902-7 (PMC6127942; doi:10.1186/s13023-018-0902-7)
Supplement: Supplementary file 1 — Interview Guide and Resources for Patients and Medical Professionals. (DOCX 44 kb) [file 13023_2018_902_MOESM1_ESM.docx]

**Online Supplementary Appendix**

**The Patient Journey to Diagnosis and Treatment of Autoinflammatory Diseases**

Jonathan Samuel Hausmann^1,2^, Kathleen Graham Lomax^3^, Ari Shapiro^4^, Karen Durrant^5^

^1^Boston Children’s Hospital, Boston, MA, USA (jonathan.hausmann@childrens.harvard.edu)

^2^Beth Israel Deaconess Medical Center, Boston, MA, USA

^3^Novartis Pharmaceuticals Corporation, East Hanover, NJ, USA (kathleen.lomax@novartis.com)

^4^Flince Research, Brooklyn, NY, USA (ashapiro@flinceresearch.com)

^5^Autoinflammatory Alliance, San Francisco, CA, USA ([karen@autoinflammatory.org](mailto:karen@autoinflammatory.org))

**Corresponding author**

Jonathan Samuel Hausmann, MD

Boston Children’s Hospital and Beth Israel Deaconess Medical Center

Boston, Massachusetts 02115

Phone: 617-355-6117

Fax: 617-730-0249

Email: [jonathan.hausmann@childrens.harvard.edu](mailto:jonathan.hausmann@childrens.harvard.edu)

**Appendix-1**

**periodic fevers patient journey research
Discussion guide**

For each discussion guide, for caregivers and, separately, for HCPs, there are two sections:

- An overall flow providing a high-level description of the session’s activities
- The detailed discussion guide, including a step-by-step outline of the conversations that the session will cover

Please bear in mind that these discussion guides describe high-level questions that researchers will explore in the sessions. Guides are flexible tools that help researchers facilitate conversations. Often, research participants share significant perspectives and insights that researchers had not previously contemplated. Aided by the guide, researchers adapt their inquiry to explore topics as they arise. As you read through these guides, please note that numbered questions (1, 2, 3, etc.) represent key themes and lines of questioning, while lettered questions (a, b, c, etc.) are probes that may or may not be appropriate given the specifics of the particular discussion.

**Interview Outline with caregivers (90-min Phone Interviews)**

| 5 minutes | Introductions |
| --- | --- |
| 10 minutes | General background   - Challenges of periodic fevers - Impact on family life - Emotional impact |
| 20 minutes | Diagnosis and initial treatment decisions   - Initial signs and symptoms - Flow through the medical system (pre-diagnosis) - Diagnosis process and its impact - Initial treatment decision-making |
| 20 minutes | Treatment experiences   - Treatment history (with a focus on colchicine and biologics) - Understanding of the treatment - Drivers of treatment initiation - Access and education - Administration, benefits, and side effects - Duration of treatment and adherence |
| 15 minutes | Relationship with medical team   - Mapping the team - Roles and responsibilities - Relationships |
| 20 minutes | Support and resources   - Education - Social / emotional support - Considerations for the future |

1. **INTRODUCTION with caregivers** (5 minutes)
   1. Moderator introduction

- 1. Confirm length of session (90-min phone interviews)
  2. Market research – no sales or promotion
  3. Audio/Videotaping
  4. Listeners
  5. Respondent confidentiality
  6. AE disclaimer
     *“Any adverse event that you raise in the course of this interview must be reported by the moderator via an Adverse Event form to local pharmaco-vigilance authorities. An Adverse Event is defined as any untoward medical occurrence in a patient administered a pharmaceutical product that does not necessarily have a causal relationship with the treatment. An adverse event can therefore be any unfavorable and unintended sign (including abnormal laboratory findings), symptom, or disease temporally associated with the use of a medicinal product, whether or not related to the medicinal product.”*
  7. **Warm-up**: To begin, please tell me a little bit about yourself and your family.
     1. Who lives with you in your household, and how are you connected (relationships)?
     2. Do you have other family or strong connections/support nearby?
     3. How long have you lived in your area/community?
     4. What kinds of activities are you and your family involved in (work, school, volunteering, clubs, etc.)?
     5. How do you manage these activities in relationship to your child’s illness?

1. **General background**  (10 minutes)

*“Let’s talk about how periodic fevers [specific fever type] has affected your life in general.”*

1. Let’s go back over the last two months. What challenges have you faced with periodic fevers, and how did you handle them?
2. Overall, what are the greatest challenges you’ve encountered in treating and managing your child’s periodic fevers on a day-to-day basis?
3. How do these challenges wax and wane between periods of fevers and other symptoms?
4. What do you do to manage these challenges before, during and after a fever episode?
   PROBE as appropriate: school attendance / performance, child’s social life, struggles over treatment, ability to work and care for child simultaneously, preparing child for independence
5. Are there aspects of your/your family’s life that have been especially affected by living with periodic fevers?
6. Are there aspects that are only minimally affected or not at all?
7. How do you feel about the impact the illness has had?
8. What kind of impact does your child’s illness have on your family’s life on a day-to-day basis?
9. Have you had to alter routines?
10. How has it impacted relationships (between both parents, siblings, etc.)?
11. Is there greater uncertainty about what will happen on a given day based on how he / she is doing?
12. Are there other impacts?
13. Do you experience any uncertainty or anxiety about how your child’s illness will change in the future and how it will affect his/her life as well as your family?
14. What are some of these concerns?
15. How do you deal with these feelings?
16. Can you think of any information or resources that might help you deal better with them?
17. **Diagnosis and initial treatment decisions**  (20 minutes)

*“Let’s talk about the process of diagnosing your child your next steps after diagnosis.”*

1. What signs or symptoms led you to seek medical care, even before your child was diagnosed? PROBE for recurrent unexplained fevers and other symptoms?

1. Did you have any idea what the cause of these symptoms could be? PROBE for family history, genetic counseling or other pre-diagnosis clues to hint at periodic fevers
2. Which physicians did you see, and why? PROBE referrals and flows through the pediatric system, especially between general PEDs and PED specialists
3. How much time passed between the first symptoms, the first doctors’ visits, and the actual diagnosis with a periodic fever? PROBE what could have been done to shorten that time?

*“Please tell me a little but about your experience surrounding the diagnosis itself.”*

1. When and how did the diagnosis happen?
2. What do you recall about it?
3. What were you told, by whom, and where?
4. What was your reaction to the diagnosis – how did you feel about it?
5. How have your thoughts about the disease changed since your child was first diagnosed?
6. How was [your child’s periodic fever] explained to you early on?
7. Was this explanation sufficient for you, or did you seek to learn more about it or clarify elements of what you were told?
8. If so, where did you look for more information, what did you learn through these efforts, and how did it change how you thought about [your child’s periodic fever]?
9. Did you enlist anyone else’s help in learning more about it?
10. If you did additional research, how well did the resources you found address your questions and concerns? Was there anything you were unable to answer, or were there any resources you wished you could use but couldn’t find?
11. What impact did the diagnosis have on your family?
12. Did your life change noticeably once the diagnosis came? How?
13. Has it changed in other ways since that early period?
14. What did you tell your child about what was happening to him / her and about the diagnosis?
15. What has been your approach to talking with him/her about it as he/she has gotten older?
16. Do you have ideas about how those conversations will evolve in the future?

*“Now let’s talk a little bit about your experiences with treatments for [your child’s periodic fever].”*

1. What did your physician tell you about the therapeutic options available to you?
2. How far into the future did this discussion go (i.e. did you discuss possible treatments to be used later down the line or just those to be used in the near term)?
3. Was there any discussion of different types of treatments (e.g. colchicine, biologics)? If so, what did you take away from these discussions?
4. What was your reaction to the discussion about treatment options?
5. Did you have any specific criteria or concerns regarding the available options (dislike of injections, worries about long-term effects, cost, etc.)? If so, how were those addressed?
6. Did you turn to any other resources in order to learn about possible treatments?
7. How helpful were these sources?
8. What did you learn from them?
9. How did you apply what you learned?
10. Do you involve your child in treatment decisions at all?
11. Would you anticipate doing so in the future?
12. **Treatment experiences**  (20 minutes)

**RESEARCHER NOTE: May need to repeat questions in this section several times, for each relevant treatment type. Pay specific attention to colchicine and any biologics (but probe on all treatments associated with periodic fevers).**

*“Let’s talk about the actual treatments that your family elected to try.”*

1. Please walk me through the history of all the different treatments you’ve tried, and when / why you started them? PROBE for entire history
2. Please explain all the transitions you made between treatments?
3. Who was involved in making each of those transitions?

**RESEARCHER NOTE: Repeat Qs 2-9 for each treatment type, as relevant. Focus on biologics and colchicine.**

1. How would you describe what this treatment does?
2. How is it different from other treatments?
3. What is your sense of how and why it works?
4. At what point did this treatment become a concrete possibility for your child?
5. Who initiated that decision (i.e. did you request it or did your physician suggest it)?
6. Did you have any concerns or unanswered questions at that point?
7. What effects (beneficial and/or negative) did you anticipate would come from making this change?
8. How did the transition to this drug happen in your case?
9. What logistical steps did you have to go through?
10. Did you encounter any obstacles in terms of access, insurance approval, or cost?
11. If so, how did you deal with them?
12. Did you reach out to anyone to learn more about starting this type of treatment, such as other families or support groups or patient advocacy organizations?
13. If so, what did you get out of those interactions?
14. How could they have been improved?
15. Have you encountered any issues with the way the medication is administered (e.g. inconvenience of infusions, difficulty with injections)?
16. If so, how have you dealt with them?
17. Have you ever considered switching to a different medication that has a different mode of administration?
18. How involved is your child in the process of administering medication?
19. What is your approach to this issue – do you feel children should be incorporated into the process, shielded from it, or some other option?
20. What are your thoughts about your child’s eventual independence and ability to care for him/herself?
21. What has been your / your child’s experience with being on these treatments?
22. The effects on your child’s health and sense of well-being, and that of your family?
23. Do you feel that they are helping your child?
24. Have there been any negative impacts (e.g. side effects) and if so how have you dealt with them?
25. What are your thoughts about how long you may be able to continue to use this treatment?
26. Will it lose its effectiveness eventually or need to be changed for some other reason?
27. Have you thought specifically about what might come after this particular treatment?
28. Do you experience any uncertainty or anxiety about the future of your child’s treatment?
29. If so, what might help alleviate those concerns?
30. Have you ever thought about stopping the medication on your own or asking your doctor about stopping? Why? What would make you stop?
31. Have you ever actually stopped the medication on your own?
32. If so, what were the reasons? [Probe reasons for stopping, why, and how, and whether the parent talked about the decision with others, and whom, as well as for danger, whether patient feels it’s actually effective, side effects, costs]
33. What would you say to fellow parents who may be considering or actually using this medication?
34. What is the most important thing for them to know or be aware of about these medications?
35. **Relationships with medical team** (15 minutes)

*“Now I’d like to talk about the doctors and medical staff that you see.”*

1. Who are the doctors and/or other therapeutic staff whom you see regularly?
2. Why do you see them, e.g. to monitor, when there is a problem, etc.?
3. How do different providers relate to one another – are they a team? Who is the leader?
4. What is the relationship like between your specialists and the general PRD? Are there ever disagreements or miscommunications between them?
5. How did you select these people to treat your child?
6. How did you find them and why did you choose them?
7. Have you ever switched doctors or other providers? If so, why?
8. How would you describe your doctors’ approach to dealing with you and to treating your child’s illness?
9. Are you happy with these approaches?
10. Is there anything you’d like to change about how you interact with your child’s doctors?
11. How would you characterize your role in making decisions about your child’s therapies – what to use, when to change, etc.?
12. Are you happy with this role?
13. Would you prefer to be more involved, or less?
14. If you’d like it to be different, what might be keeping you from assuming your preferred role?
15. Do you feel you adequately understand what your medical team tells you about [your child’s periodic fever] and its treatment?
16. Have there been specific occasions where you have felt confused or lacking information/explanation?
17. How have you handled these situations?
18. Do you talk to other people or use other resources to try to clarify what you hear from your medical team? If so, where do you turn?
19. **Support and resources** (20 minutes)

*“Let’s talk some more about how you have learned about the disease and its treatments.”*

1. How important do you feel it is for you to know a lot about [your child’s periodic fever], treatments, etc.?
2. Can it be left to the medical team, or do you need to figure it out yourself as well?
3. If you do want to know, how does that knowledge help you?
4. If you do want to learn more, are there some sources that are better than others? What are some examples?
5. What makes these sources good – easier to understand, more complete, more reliable / trustworthy, etc.?
6. Have you ever received a communication (like a flyer) or looked at a resource (like a website) that really turned you off? If so, what was off-putting about it? Why didn’t you like it?
7. How about a specific source that you really liked – what was good about it?
8. Are there topics or issues where you feel like you lack adequate information? If so, what are they?
9. Have you made efforts to learn and been unsuccessful? If so, what have you tried?
10. How do you think knowing more might help you?
11. Have you ever turned to someone outside your immediate family for support in dealing with [your child’s periodic fever]? To whom have you turned, and how did they help you?
12. Have you ever used, or thought about using, a support group (live or online) to help deal with managing [your child’s periodic fever]?
13. If so, what has that experience been like?
14. If not, what do you think about the idea of doing so?
15. How do you feel about your child’s future with this disease?
16. How will your child’s health change, and what impact do you foresee it having on your family’s life in general?
17. How much do you think about issues like this?
18. How do you deal with the emotional impact of thinking about it?

**RESEARCHER NOTE: Review diary and video camera, review patient drawing exercise, discuss timeline for completion and any other open questions or concerns. Thank and end.**

**periodic fevers patient journey research
HCP Discussion guide**

**Interview Outline (60-min):**

| 10 minutes | 1. Introduction  - Introduce research session - Physician practice/background - Involvement in periodic fevers and their treatment |
| --- | --- |
| 25 minutes | 1. Periodic fevers and their treatments  - Diagnosis and referral patterns - Treatment options and decision-making - Coordination of care (specialists, centers, etc.) |
| 10 minutes | 1. Expectations for the future of therapy  - Expectations regarding the potential of new therapies - Awareness/experience with biologic agents - Projected therapeutic landscape in 2018 |

1. **INTRODUCTION with HCPs** (10 minutes)
   1. Moderator introduction
   2. Confirm length of session (60 minutes)
   3. Market research – no sales or promotion
   4. No vested interest in any particular outcome
   5. Confidentiality – No names in report
      (I’m interested in what you say, not who says it)
   6. Audio taping / recording
   7. Respondent confidentiality
   8. AE disclaimer
   9. **Objectives**: The focus of this research session is to review treatment practices for patients with periodic fevers, focusing on FMF, TRAPS, and HIDS.
   10. PHYSICIAN BACKGROUND and practice characteristics:
       - Type/location of practice
       - Patient census
       - Number of periodic fevers patients overall
       - Number of FMF, TRAPS, and HIDS patients
   11. Please tell me a little more about how you became involved in the care of patients with periodic fevers…
   12. How long have you been involved in caring for periodic fevers patients?
   13. How did you get involved in this therapeutic area?
   14. What proportion of the patients you see have periodic fevers?
   15. What proportion of those have FMF, TRAPS, and HIDS in particular?
   16. How would you characterize the difference between these various conditions?
   17. Are there differences between treating FMF, TRAPS, and HIDS patients?
   18. What are the biggest challenges you face in working with these patients and families in particular?
   19. What are your greatest rewards in working with these patients and families?
2. **Periodic fevers & their treatment** (25 minutes)
3. Please estimate the incidence and prevalence of periodic fevers in your community? PROBE
   1. Are they associated with a specific ethnic group?
   2. Are they more prominent in your community vs. elsewhere in the US?
   3. Do you see a disproportionate number of these patients relative to other [specialists] in your community
4. Please tell me a bit about the diagnosis of patients with periodic fevers?
   1. How simple/complicated is the diagnosis of periodic fevers?
   2. What are the typical steps in the diagnosis process?
   3. To what extent is the disease diagnosed differentially based on symptoms, vs. genetic testing?
   4. How long / short is the typical time to diagnosis (and how much variability do you see)?
5. Who typically diagnoses patients with periodic fevers?
   1. What is the typical referral pattern for these patients?
   2. At what point do patients end up in your office? Why?
   3. And when do you refer them on to other HCPs?

PROBE: different physician specialists, different institutions, community vs. tertiary / center of excellence

1. In your experience, what causes delays to diagnosis, missed or misdiagnoses, and other issues?
   1. What do you see as bottlenecks / areas of unmet needs?
2. How (if at all) is diagnosis different for patients with varying types of periodic fevers (focusing on FMF, TRAPS, and HIDS)?
3. Tell me about referral processes and the flow of patients between specialists, after the diagnosis is made? PROBE
   1. How are the following specialists involved: Rheumatologists, Dermatologists, Neurologists, others?
   2. How and why do patients move between the community and tertiary centers?
   3. Who is ultimately responsible for the patient / family, and why?
4. Please tell me about a typical initial (first) visit with a patient / family....
   1. What information is typically reviewed?
   2. What additional tests do you typically order?
   3. What therapeutic decisions are typically made at that point in time?
5. What role do patient-specific characteristics play in your therapeutic decision-making for periodic fevers? PROBE
   1. Frequency or severity of the fevers?
   2. Presence / absence of other symptoms? Which ones?
   3. Age?
   4. Social / family situation?
   5. Other factors?
6. How do these characteristics drive specific treatment decisions for patients with periodic fevers? PROBE
   1. When/why is colchicine indicated? PROBE what symptoms drive you to initiate that drug?
   2. When/why are biologic agents used? PROBE Unaided for any use of anti-TNFs, Kineret, Ilaris?
7. What role do families’ stated desires for therapy play in driving treatment decisions? PROBE
   1. How do you see these diseases impacting families: emotionally, practically, logistically?
   2. What common challenges do these families face, and what victories do they share?
   3. How do their experiences at home impact their thinking about whether, when and how to treat these diseases?
8. What role do specific reimbursement considerations play in your treatment options? PROBE
   1. How do you code these patients for well / sick visits?
   2. How well / poorly do the requirements of ICD codes and insurance requirements support the optimal management of these patients?
9. Are there guidelines that inform your treatment algorithm? Institutional? Region? National? International?

1. How do you monitor periodic fever patients in between periods of illness?
   1. How frequently do you see patients in between periods of illness?
   2. What scans/tests to do you order and why?
   3. What are you looking for?
2. What emerge as issues in the long-term care of periodic fever patients? PROBE
   1. Other symptoms including autoimmune and neurological issues?
   2. Long-term impacts of parents, siblings, families, etc.
3. What do you see as the natural history of periodic fevers?
   1. In your experience, do children tend to develop along a specific timeline or trajectory?
   2. How does this differ amongst the various fevers (focus on FMF, TRAPS, and HIDS)?
4. Before we move into the next part of our discussion, what other issues should we discuss relative to the current state of the art in treating patients with periodic fevers generally? FMF, TRAPS, and HIDS in specific?

**C. Expectations for the future of therapy** (10 minutes)

*“Now, I’d like to shift our conversation to the future of therapy for kids with periodic fevers.”*

1. What changes do you anticipate in the treatment of these diseases? In the next year or two? In the next five or ten years? Where do you see the field going? Why?
   1. Changes in drug therapy?
   2. Changes in approaches to coordinated care / management?
2. What does the ideal treatment for this condition look like? What would you like to see emerge, and why?
3. Do you see any (additional) opportunities for treating these diseases with biologic therapy? PROBE
   1. How?
   2. Why?
4. Please summarize what you know about any new / pipeline drug therapies for these diseases? PROBE Efficacy data; safety / side effects data
   1. Have you used these drugs in your clinical practice?
   2. What are your impressions of the drugs based on clinical experience?
   3. What do you hear from colleagues / KOLs about them?
5. What do you see as the future of anti-IL-1b drugs in this disease?
   1. To what extent have you used anti-IL-1b drugs in clinical practice to date?
   2. What do you see as the clinical prospects for these therapies in periodic fevers? Why?

**Appendix -2**

**Helpful resources for medical professionals and patients:**

*The EUROFEVER Classification* Criteria online tool to evaluate symptoms, when considering genetic testing for a patient. http://www.printo.it/eurofever/scoreCriteria.asp

*The Registry of Hereditary Auto-inflammatory Disorders Mutations is* one of many resources to help consider specific mutations for various autoinflammatory diseases.
<http://fmf.igh.cnrs.fr/ISSAID/infevers>

*Dermatology Online Image Atlas* - DermIS Dermatology Information System has a wealth of information and images for autoinflammatory diseases.
<http://www.dermis.net/dermisroot/en/home/index.htm>
 *Autoinflammatory-search.org* from the Autoinflammatory Alliance is a comparative, medically edited database/chart of autoinflammatory diseases that is searchable, with hyperlinked journal articles for all the listed symptoms for each autoinflammatory disease, along with photos of rashes and genetic testing links. This site has been used by doctors to compare symptoms, to educate, and help doctors to consider these diseases in their patients. <http://www.autoinflammatory-search.org/diseases>

*Diagnostic Tools for Autoinflammatory Diseases*, includes a downloadable pdf table of US genetic testing panel options, and links other helpful sites that can help medical professionals that are seeking to do genetic testing on their patients for autoinflammatory diseases. <http://saidsupport.org/diagnosis-genetics-periodic-fever-syndromes/>

*Autoinflammatorydiseases.org* is a blog written by a pediatric and adult rheumatologist with an interest in autoinflammatory diseases. Posts include cutting-edge disease updates, videos about managing AIDs, symptom tracker PDFs, and background information about AIDs helpful to both patients, families, and physicians.

**Helpful resources for patients:**

*The Autoinflammatory Alliance* features many resources online, and in print for medical professionals, patients and families, especially those that are trying to navigate the process of diagnosis, and treatment for periodic fever syndromes (autoinflammatory diseases) on <http://autoinflammatory.org> and their blog at . <http://saidsupport.org>. Some top resources include:

1. *My Child, or I Keep Getting Fevers. What Do I Do?* This saidsupport.org blog post with links to various pages of information has helped a lot of patients and families to have a plan to track symptoms, common tests ordered by doctors to evaluate patients for these diseases, and how to complile your medical records and symptom logs for appointments. <http://saidsupport.org/child-keeps-getting-fevers>
2. *Learn* is a basic introduction to autoinflammatory diseases, with more information about specific diseases is on the main Autoinflammatory Alliance website at: <http://autoinflammatory.org/learn_intro.php> and also some downloadable materials are on the site about certain diseases.
3. *Healthier Ever After,* is a book and injection tips guide and rewards program for children that need to take injectable biologic medications for their autoinflammatory disease. The book is on this link as a pdf, and also available in pdf and print in a number of languages. Contact the Autoinflammatory Alliance for other languages. <http://saidsupport.org/debut-healthier-ever-injection-tips-book-autoinflammatory-diseases/>
4. The Autoinflammatory Alliance has autoinflammatory disease information cards that are medically edited, business card sized documents that have key considerations needed for many of the autoinflammatory diseases, with one card per disease. These are available to be mailed to patients or medical professionals, and are helpful for patients to have in their wallet to give to doctors in the ER, or stable to school forms to explain each disease. Contact [Karen@autoinflammatory.org](mailto:Karen@autoinflammatory.org) to request these complimentary cards.
5. *School 504 Plans for Kids with Periodic Fever Syndromes* is on the saidsupport.org blog, and also as a downloadbable pdf for families that are needing accommodations in their child’s school due to an autoinflammatory disease. <http://saidsupport.org/school-504-plans-for-kids-with-periodic-fever-syndromes/> *Supporting Children with Periodic Fever Syndromes at Schools in the UK.* This is the UK version of the accommodations guide for children with autoinflammatory diseases in UK schools. <http://saidsupport.org/supporting-children-with-periodic-fever-syndromes-at-schools-in-the-u-k/>

*PReS/SHARE Printo.it Information on Pediatric Rheumatic Diseases* Autoinflammatory disease pdfs that can be viewed, and printed in many languages are available at: <https://www.printo.it/pediatric-rheumatology/GB/info/sub/11/Autoinflammatory-Diseases>

*Patient Information Guides* downloadable pdfs for many autoinflammatory diseases are available for patients from The Periodic Fever Service at the National Amyloidosis Centre in the UK:

<http://www.amyloidosis.org.uk/fever-syndromes/the-inherited-fever-syndromes-information-on-each-syndrome/>

*Living with Periodic Fevers* is a website sponsored by Novartis that has a lot of helpful information for patients and caregivers, and even some children’s storybooks at: <http://periodicfevers.com/>

*RareConnect*, hosted by EURORDIS has some communities for autoinflammatory diseases with moderators from various organizations. <https://www.rareconnect.org/en> There are also other private patient groups online on various social media platforms.
